# Quarterly Report (No. 3,) of the Medical Diseases Affecting Children, Treated at the Royal Infirmary for Sick Children, from the 10th of March to the 10th of June, 1823

**Published:** 1823-07

**Authors:** John Webster

**Affiliations:** one of the Physicians to that Institution.


### STATISTICAL MEDICINE.

ART. I.—*Quarterly Report (No. 3,) of the Medical Diseases affecting Children, treated at the Royal Infirmary for Sick Children, from the* 10*th of March to the* 10*th of June,* 1823. By JOHN WEBSTER, M.D. one of the Physicians to that Institution.

*Diseases affecting particular Organs.*

| | | | | |
|---|---|---|---|---|
| Head and Nervous System | | Hydrocephalus Acutus | 17 | |
| | | Epilepsia | 1 | |
| | | Deliquium Animi | 1 | = 19 |
| Organs of Respiration | | Hæmoptisis | 1 | |
| | | Catarrhus | 2 | |
| | | Bronchitis | 38 | |
| | | Tussis | 3 | |
| | | Pleuritis | 2 | |
| | | Pneumonia. | 9 | |
| | | Pertussis | 29 | |
| | | Phthisis | 4 | = 88 |
| Organs of Digestion | Mouth, &c. | Aptha | 11 | |
| | | Cynanche Parotidea | 4 | |
| | | ——— Tonsillaris | 7 | |
| | Stomach | Dyspepsia | 13 | |
| | Bowels | Constipatio | 11 | |
| | | Diarrhœa | 16 | |
| | | Dysenteria | 11 | |
| | | Enteritis | 1 | |
| | Liver | Hepatitis | 1 | |
| | All three | Cholera | 14 | |
| | Spleen | Splenitis Chronica | 1 | |
| | General | Marasmus | 2 | |
| | | Colica Pictonum | 1 | |
| | Worms | Lumbricus | 1 | |
| | | Ascarides | 8 | =102 |
| Skin Eruptions | Acute | Variola Confluens | 2 | |
| | | Variola Discreta | 13 | |
| | | Varicella | 3 | |
| | | Morbilli | 3 | |
| | | Rubeola | 1 | |
| | | Milliaria | 7 | |
| | | Urticaria | 3 | |
| | | Scarlatina | 5 | |
| | | Pompholyx | 1 | |
| | | Strophulus | 1 | |
| | | Purpura | 1 | |
| | Chronic | Psoriasis | 2 | |
| | | Impetigo | 3 | = 45 |

*Diseases affecting various Parts.*

| | | | |
|---|---|---|---|
| Fevers | Continua Simplex | 7 | |
| | ——— Cephalica | 11 | |
| | ——— Pleuritica | 11 | |
| | ——— Gastro-enteritica | 53 | |
| | Remittens | 38 | |
| | Intermittens, Tertiana | 1 | =121 |
| Cachexia | | 6 | |
| Vaccinated | | 11 | |
| Swallowed a piece of iron, of 158 grains weight | | 1 | = 18 |
| | Total number of patients | | 393 |

*Fatal Cases reported at the Infirmary:*—Hydrocephalus, 2; Bronchitis, 1; Pneumonia, 2; Phthisis, 2.—Total, 7.

The principal features in the present Report, compared with that of the preceding quarter,* are the great diminution in the number of diseases affecting the organs of respiration; whilst those of the digestive organs, fevers, and particularly eruptive complaints, have very much increased. At the same time it may be remarked, that, in general, diseases, with the exception of a single class, have been of a milder type than they were during the winter; when affections, especially of the respiratory organs, were unusually frequent and severe.

The exception above alluded to comprehends Hydrocephalus, of which seventeen cases were met with during the period embraced in the present report, and most of them were of an exceedingly acute character; so much so as to make it, in the opinion of the author, worthy of being mentioned. Two fatal instances occurred; one of them a relapse, when the child had nearly got well. In this case the body was examined, and upwards of six ounces of serum were found in the ventricles. After bleeding, purging, and the warm bath, the most efficacious medicines in the treatment of hydrocephalus were diuretics, which *then* appeared to produce most decided effects, quite consonant to the character already given of them in a previous report.

Of the seven fatal cases reported, five were examined: only one of these presented appearances of sufficient interest to deserve particular description. The subject of it was five months old, and never had enjoyed perfect health from its birth, having a large belly, and always either disordered in its bowels or suffering from affections of the organs of respiration. When first seen, the infant was labouring under an attack of cholera, which soon gave way to the appropriate remedies; but this was followed by a new accession of inflammation of the lungs, from the effects of which the little patient never recovered, but died in a short time after, quite hectic. On opening the cranium, the veins on the surface of the brain were slightly congested, and an effusion of lymph was observed under the pia mater in several places; the hemispheres adhered to each other, and about an ounce of serum was found in the ventricles. At the conjunction of the optic nerves, a large ulcer was discovered, which burrowed into the substance of the right anterior lobe of the brain. On cutting into the pons varoli, several tubercles, four at least, were met with, full of a cheesy-like matter, the largest of them being about the size of a small swan-shot. The thymus gland was much enlarged, and in a state of ulceration. The larynx, trachea, and bronchia, were very much inflamed; as also the substance of the lungs, which was quite filled with congeries of tubercles, many of them in a suppurating state. The left lobe of the lungs was hepatized, and a large ulcer was discovered in the upper part of it, with great destruction of substance. The diaphragm was studded all over with small spots, very similar in appearance to the tubercles in the lungs; several in the substance of the liver; and the spleen was also quite full of them; in-

* See the Number of this Journal for April.

numerable mesenteric glands, greatly enlarged, with most of them in a complete state of ulceration; and the mucous coat of the small intestines inflamed, and abraded at many points. The kidneys were free from any marks of disease.

---

### METEOROLOGICAL JOURNAL,

